# Accuracy of a Smart Diaper System for Nursing Home Residents for Automatically Detecting Voided Volume: Instrument Validation Study

**DOI:** 10.2196/58583

**Published:** 2024-10-24

**Authors:** Jae Heon Kim, Ui Cheol Lee, Byeong Hun Jeong, Byeong Uk Kang, Sung Ryul Shim, In Gab Jeong

**Affiliations:** 1 Department of Urology Soonchunhyang University Seoul Hospital Soonchunhyang University College of Medicine Seoul Republic of Korea; 2 HYGERA Networks Inc Seoul Republic of Korea; 3 Department of Biomedical Informatics College of Medicine Konyang University Daejeon Republic of Korea; 4 Konyang Medical Data Research Group KYMERA Konyang University Hospital Daejeon Republic of Korea; 5 Department of Urology Asan Medical Center University of Ulsan College of Medicine Seoul Republic of Korea

**Keywords:** smart diaper, urinary incontinence, medical device, voided volume, urine output, nursing home, older adults

## Abstract

**Background:**

Diapers are commonly used by older patients with urination disorders. A smart diaper system (SDS) may be able to estimate the weight of urine comparably to conventional measurements made by weighing diapers.

**Objective:**

The aim of the current research is to determine the degree of accuracy of an SDS technology specifically designed for the management of urination routines and the use of incontinence pads in older adults.

**Methods:**

From January to December 2022, 97 older patients with at least 1 chronic disease from 3 nursing homes were included. In this study, the SDS was used for 1 month per patient after obtaining their consent; all patients originally used traditional diapers in the nursing home. The index test measured the change in electrical resistance of the SDS and the reference test measured the change in actual urine weight. When measuring the actual urine weight, the degree of absorption was directly confirmed with the naked eye because the expression value varied according to pressure changes caused by the user’s movement or position. The Pearson correlation was used to determine the correlation between the 2 test methods, the intraclass correlation coefficient (ICC) was used to check the degree of agreement between the evaluators, and the Bland-Altman test was used to confirm whether there was a significant difference between the 2 test methods.

**Results:**

The average age of the 97 participants was 86.48 (SD 6.26) years, with 10 men and 87 women. There were 73 patients (75%) with hypertension, 86 patients (88%) with dementia, and 86 patients (88%) with 2 or more comorbidities, accounting for the majority. The Pearson correlation coefficient and ICC were 0.971 and 0.985 (*P*<.001). In the Bland-Altman figure, the difference in the mean between the 2 tests was evenly scattered without showing a specific pattern, indicating that the SDS and actual urine weight were very consistent. The difference between the mean of the 2 tests was –0.045 of the standardized mean difference, and all measurements were located within the 95% CI, so this confirms that the 2 test methods are equivalent.

**Conclusions:**

Our study showed a fairly high correlation coefficient and ICC for all patient groups, which reveals that the 2 tests were very consistent and that the SDS can replace traditional diapers, even in a real clinical setting. This study shows the possibility that heath care professionals could be alerted by the SDS to the need for pad replacement due to incontinence, thus avoiding the development of dermatological complications.

## Introduction

In high-income nations, there is a gradual trend toward a longer human lifespan. However, this is accompanied by a number of age-related issues, such as bladder dysfunction, which causes lower urinary tract symptoms (LUTS) [1]. The latter can be classified into three groups: (1) storage, or irritative, symptoms, such as urgency, frequency, nocturia, and urge incontinence, which together are referred to as overactive bladder syndrome; (2) voiding or obstructive issues, including a diminished stream strength, hesitancy, and incomplete bladder emptying and straining; and (3) problems occurring following micturition, which encompass a sensation of retained urine and dribbling after urination [1].

In older people, LUTS impact the ability to perform activities of daily living, reduce confidence levels, and lead to a loss of mobility and a greater likelihood of requiring inpatient care [2]. Within populations, urine infections occur with a frequency between 5% and 45% [3,4], with age representing a significant risk factor [5,6]. Incontinence is twice as common in individuals cared for in nursing homes as in those in the community; the prevalence in the former is over 50% [7].

Older people, and particularly those who are frail, frequently have bladder dysfunction. Consequently, the use of absorptive incontinence pads or indwelling bladder catheterization is often required to manage LUTS [8]. The use of incontinence pads is not without risk. For instance, regular checks on the state of the pads, especially during the night, may cause patients discomfort, disrupt their sleep patterns, or interfere with their routine daily activities [9]. Prolonged contact with a soiled pad may also induce additional comorbidities, such as skin inflammation and infection with bacterial pathogens [10,11].

Incontinence pads require frequent replacement, which is a labor-intensive task. The aim of the current research is to determine the degree of accuracy of technology that has been specifically designed for the management of urination routines and the use of incontinence pads in older adults. It comprises a dedicated sensor incorporated into an incontinence pad, in other words, a smart diaper system (SDS) that has the ability to recognize bladder voiding and to quantify the urine volume produced. Determination of the practicality and application of this technique as a substitute for traditional incontinence pads in the clinical arena was the objective of this study.

## Methods

### Patients and Methods

The Strengthening the Reporting of Observational Studies in Epidemiology (STROBE) guidelines were followed in this study (Multimedia Appendix 1) [12]. This study was conducted in Korea between January 1, 2022, and December 30, 2022, among residents at the Seoul Southern Senior Nursing Home in Gunpo, Gyeonggi-do, a nursing home in Hwaseong, Gyeonggi-do, and the Namyangju Veterans Nursing Home in Namyangju, Gyeonggi-do, all of which use an SDS. This study included patients with at least 1 of the following conditions: hypertension, diabetes, and dementia. In this study, the SDS was used for 1 month per patient after obtaining consent; all patients had mobility difficulties within the nursing homes and originally used a traditional diaper. Information on the patients’ gender, diseases, degree of convulsion, BMI, and meal pattern was collected. The SDS users were mainly women. In the case of men, the frequency of SDS use was low, and some data were missing because they did not use the SDS continuously on a daily basis, so it was necessary to compare data continuity. Among the study participants, those who disagreed with the study or were uncomfortable with the SDS were excluded. All patients who participated in this study had poor mobility and at least 1 disease, but they participated in the study after the purpose of their participation was explained.

### Ethical Considerations

This was an observational study of patients who already used diapers, so there was no expected harm to the patients; also, the additional research activities required only measuring the actual weight of urine, so there were no ethical concerns related to the patients’ participation. Informed consent was obtained and this study was fully explained to patients who participated. This study was approved as a retrospective analysis of continuous monitoring data on voiding patterns obtained from diapers equipped with a smart sensor for older people with disabilities; approval was granted by the Institutional Review Board of Soonchunhyang University Seoul Hospital (2023-02-013).

### Outcome Measurements

This research ultimately aims to monitor the amount of urine per urination and the total amount of urine per day. As a starting step, this study compared the accuracy of SDS measurements of the amount of urine per urination and the actual weight. When measuring the actual urine weight, the degree of absorption was directly confirmed with the naked eye because the expression value varied according to pressure changes with changes in the user’s movement or position. The weight of urine was determined by measuring the total weight of the diaper using an electronic scale and then calculating the weight difference with an unused diaper. In addition, the measurer was a person with a nursing care worker’s license and the data were collected by a researcher with more than 1 year of work experience.

**Figure 1:**
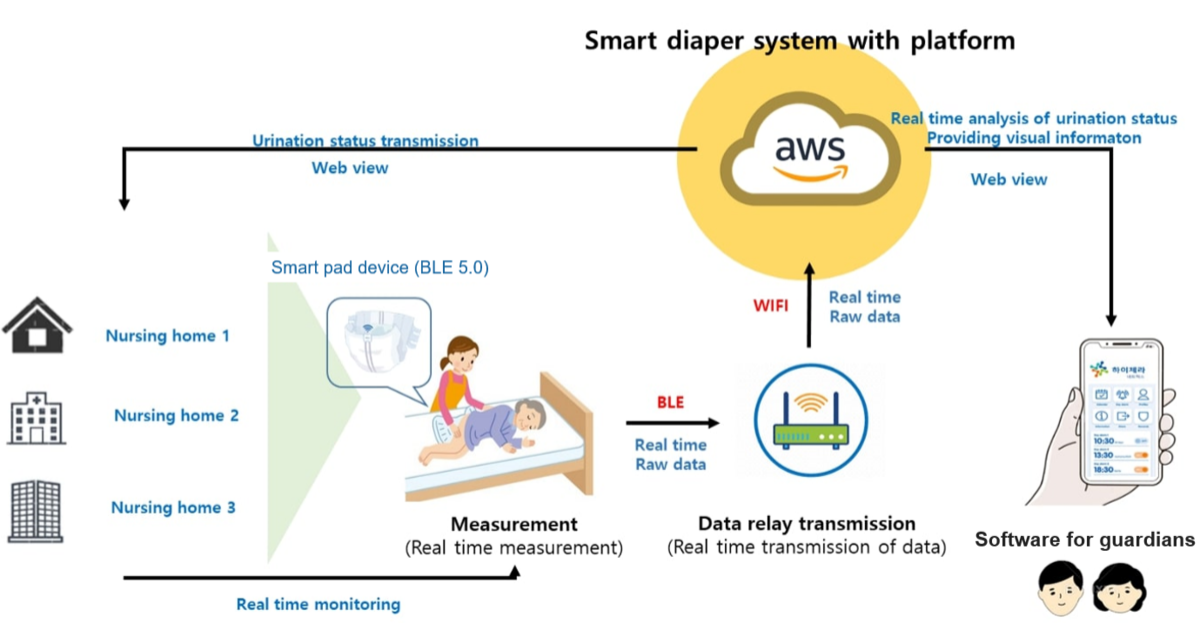
Composition of smart diaper system. The smartphone app transmits urination detection information to AWS (Amazon Web Services) in real time. BLE: Bluetooth Low Energy.

**Figure 2:**
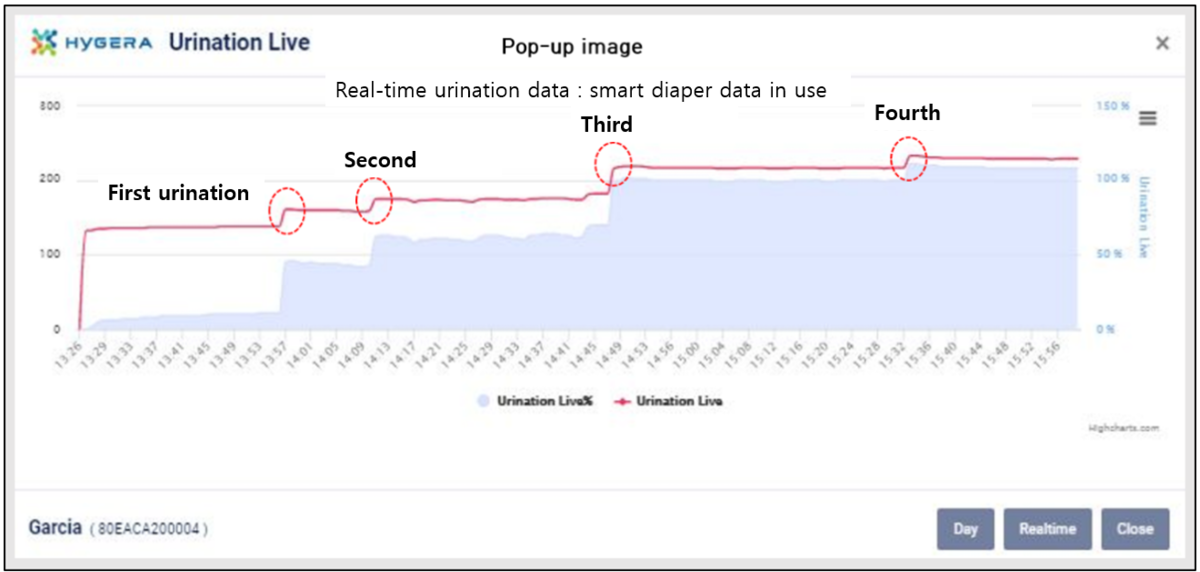
Urination data chart of a single smart diaper. Wetness is detected every 15 seconds. Urination judgment follows an artificial intelligence algorithm.

The urination detection and urination volume calculation methods were as follows: When moisture came into contact with 2 parallel pairs of conductive material mounted inside the diaper, the electrical resistance was detected every 15 seconds, and the detected information was transmitted to a smartphone app or wireless repeater. For urination measurement, the diaper wetness was set to the minimum and maximum saturation (from 3 ml to 500 ml); measurements from the SDS were converted to percentiles, and the amount of urine was then calculated as its weight. The smartphone app transmitted urination detection information to AWS (Amazon Web Services) in real time and displayed the transmitted data in real time as a chart (Figure 1). This chart visualized the urination state in the diaper every 15 seconds. The chart was created regardless of whether or not there was a change in wetness. The urination state was determined by artificial intelligence based on the chart (Figure 2); the visualization information was provided through a smartphone or PC.

### Statistical Analysis

Data were analyzed using R (version 4.3.1; R Foundation for Statistical Computing), and participants’ general characteristics were analyzed using frequency analysis. The index test measured the change in electrical resistance of the SDS and the reference test measured the change in actual urine weight. The Pearson correlation coefficient is widely used to linearize values between pairs of test methods and confirm their correlation [13]; it is a value obtained by dividing the covariance of 2 variables by the product of their respective SDs in the data of the interval scale or proportional scale, which accurately shows the correlation between the 2 tests. Pearson correlation analysis was used to determine the agreement between the SDS measurements and urine weight change. The intraclass correlation coefficient (ICC) was calculated to confirm the reproducibility of the comparator and reference tests. ICCs are an estimate of the portion of the total variation in the measurements caused by individual variation and have values between 0 (not at all matched) and 1 (perfectly matched) [14]. ICCs can also be considered as representing the interchangeability of evaluators by confirming the agreement between them in a reliability study that considers the effect of evaluators in the fluctuation of measured values. As an indicator of agreement, the ICC is considered to be a better indicator than correlation coefficients because it contains information on both correlation and bias between measured values [15].

The Bland-Altman test calculates the mean of and difference between each pair of measurements from the 2 tests and represents the mean on the x-axis and the difference on the y-axis; it is very useful not only in repeatability and reproducibility evaluation, but also in examining the disparity between measurements by pairs of different test methods, so it is widely used in comparative studies of test methods [16,17]. For the Bland-Altman test, since the actual amount of change in urine weight and the amount of change in electrical resistance measured by the SDS had different units of measurement, each of the measured values was divided by the SD and standardized to minimize bias from comparator tests [18]. All statistics were 2-tailed, and *P* values <.05 were considered significant.

## Results

### General Characteristics

The average age of the 97 participants was 86.48 (SD 6.26) years, with 10 men and 87 women. There were 73 (75%) patients with hypertension, 86 (89%) patients with dementia, and 86 (89%) patients with 2 or more comorbidities, accounting for the majority (Table 1).

**Table 1:** Participant characteristics (N=97).

Characteristics		Values
Age (years), mean (SD)		86.48 (6.26)
**Sex, n (%)**		
	Male	10 (10)
	Female	87 (90)
Weight (kg), mean (SD)		45.88 (8.14)
BMI (kg/m^3^), mean (SD)		19.74 (2.84)
**Number of complications, n (%)**		
	1	11 (11)
	2	44 (45)
	3	30 (31)
	4	12 (12)
Hypertension, n (%)		73 (75)
Dementia, n (%)		86 (89)
Index test_change R, mean (SD)		209.16 (94.41)
Reference test_change unrine weight, mean (SD)		208.35 (92.08)

### Correlation and ICC Between SDS and Urine Weight

**Figure 3:**
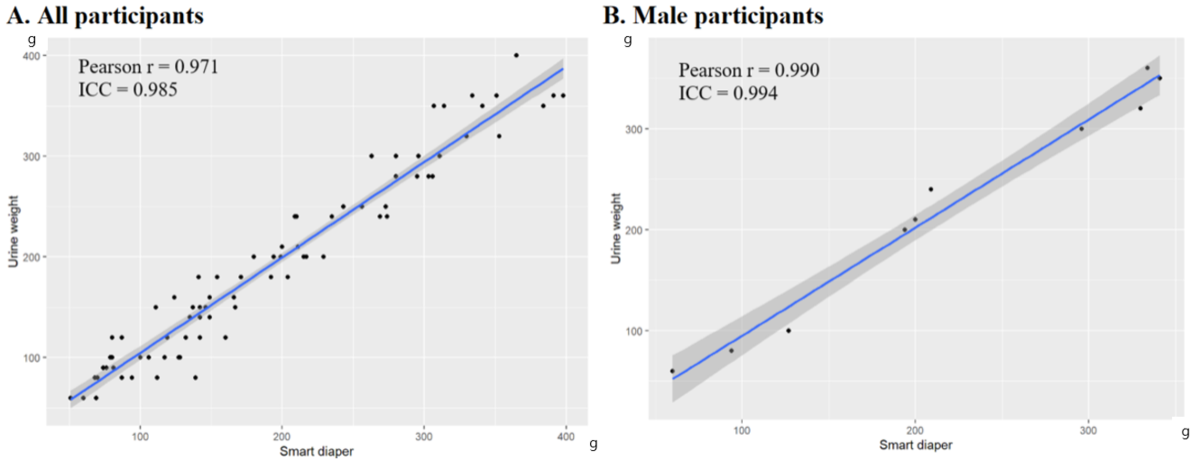
ICC (intraclass correlation coefficient) between smart diaper measurements and actual urine weight in all participants (A) and male participants (B).

The Pearson correlation coefficient in all participant groups was 0.971 (*P*<.001). In the group with more than 3 complications, the Pearson correlation coefficient was 0.971, and in the male and female groups, it was 0.990 and 0.969, respectively (all *P*<.001). In all groups, there was a very strong correlation between the 2 tests (Table 2 and Figure 3). ICC in all participant groups was 0.985 (*P*<.001). In the group with more than 3 complications, the Pearson correlation coefficient was 0.985, and in the male and female groups, it was 0.994 and 0.984, respectively (all *P*<.001). In all groups, there was a very strong reproducibility between the 2 tests (Table 2 and Figure 3).

**Table 2:** Association between index test and reference test for measuring urine weight changes.

Specific groups	*r*	*P* value	ICC^a^	*P* value
All participants (N=97)	0.971	<.001	0.985	<.001
Hypertension (n=73)	0.967	<.001	0.983	<.001
Dementia (n=86)	0.970	<.001	0.985	<.001
More than 3 complications (n=42)	0.971	<.001	0.985	<.001
Male (n=10)	0.990	<.001	0.994	<.001
Female (n=87)	0.969	<.001	0.984	<.001

^a^ICC: intraclass correlation coefficient.

### Bland-Altman Test Comparing SDS and Urine Weight

**Figure 4:**
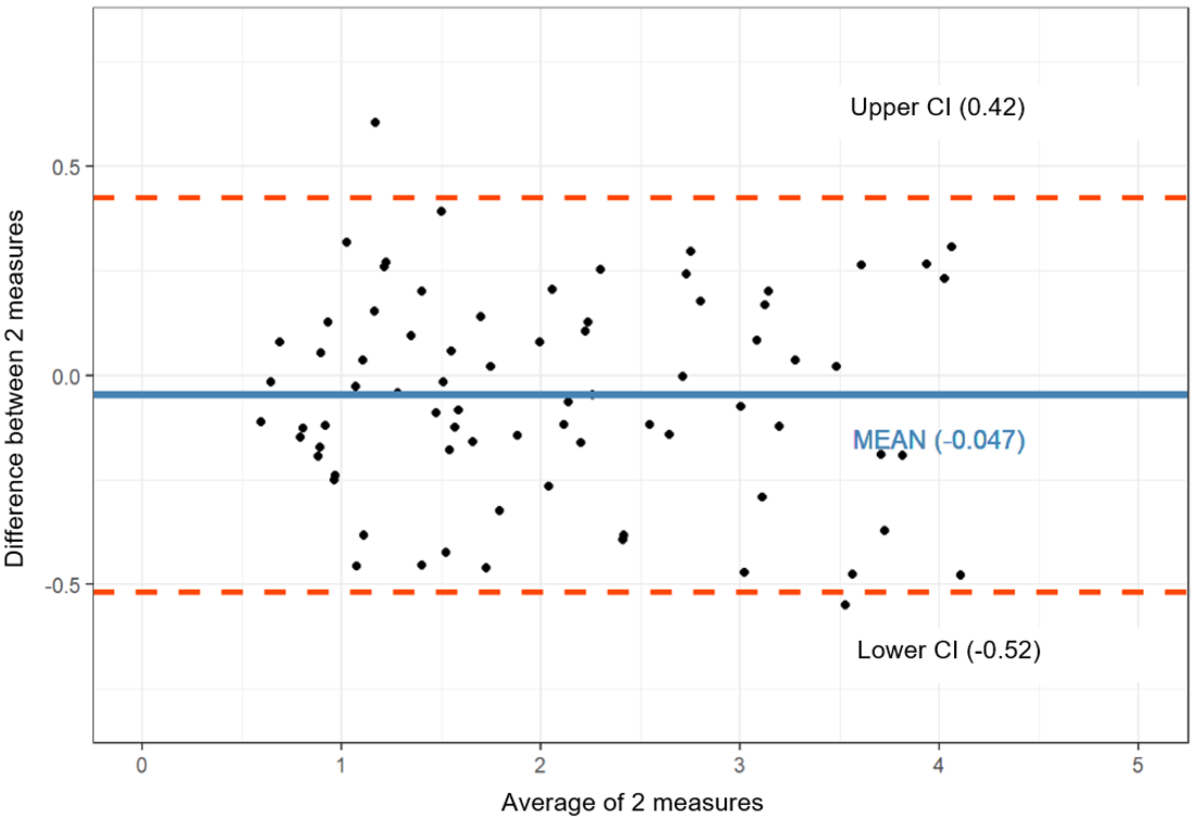
Bland-Altman plot showing average and difference between the 2 measures (the smart diaper system and actual urine weight). The middle solid line shows the mean and the top and bottom dotted lines show the 95% CI.

The Bland-Altman plot (Figure 4) identified 3 kinds of agreement: distribution, mean difference, and limit of agreement (LOA). First, as the value of the x-axis (standardized urine weight) increases, the difference in the mean between the 2 tests is evenly scattered without showing a specific pattern, indicating that the 2 tests were very consistent. Second, the difference between the mean of the 2 groups was –0.045 of the standardized mean difference (SMD), which is about 2.6% of the effect size. This is a small number that can be ignored in the actual urine weight measurement, so it can be seen that the 2 tests were also very consistent. Finally, all measurements were located within the 95% CI (in LOAs), so this confirms that the 2 test methods are interchangeable.

## Discussion

### Principal Results

The results of this study indicate that the SDS, which was designed using Internet of Things (IoT) technology, could be successfully implemented in real-world scenarios, such as patients being cared for in a hospital. Patients admitted for acute medical management require accurate fluid and electrolyte monitoring, which calls for routine urine output measurements to be collected. We found that the volume measurements obtained using the SDS sensor showed similar precision to those obtained with the traditional method of weighing the patient’s wet pad. Our study showed a fairly high correlation coefficient for all patient groups, which reveals that the 2 tests were very consistent and that SDS can replace traditional diapers, even in a real clinical setting. In addition, the high ICC also showed that the degree of agreement among measurers was high, indicating that SDS is a reliable measurement tool in any clinical environment.

IoT technology has advanced extremely quickly, facilitating a number of changes in intelligent clinical practices. Progressively more conventional health care disciplines are being supported with or substituted by IoT techniques [19]. Examples include the development of the SDS, which involves the use of wireless technologies and diminutive sensors that can be used by patients in an incontinence pad. This enables the presence of moisture in absorptive materials to be recognized and can alert health care providers that the incontinence pad requires changing, as well as facilitating concurrent urine output measurements [20,21].

### Comparison With Prior Work

The utility of an SDS for the recognition of urination and urine volume measurement was assessed by Cho et al [22] in relation to its impact on the skin inflammation associated with urine incontinence in an acute care facility. The previous study included 30 patients, and 390 incidences of urine voiding were documented. The number of records acquired using frequency volume charts (FVCs) and the SDS were as follows: 108/390 (27.7%) for both FVC and SDS, 258/390 (66.2%) for FVC, 18/390 (4.6%) for SDS, and 6/390 (1.5%) for FVC (with SDS lost). The SDS exhibited a detection frequency of 32.8%. Where data were available from both FVCs and the SDS, a strong correlation was demonstrated (*R*^2^=0.88; *P*<.001). These authors were the first group to evaluate the application of a SDS based on Bluetooth and a smartphone in this context. The accuracy, although not excellent, was deemed sufficient for clinical use.

A further study assessed the use of an external SDS in 18 patients with dementia in 3 different nursing homes [23]. The SDS alerted heath care providers to the presence of urine and the requirement for an incontinence pad change. However, the measurements obtained from the sensor and those recorded on the FVCs failed to correlate, with the sensor findings being 26% and 34% lower and 39% and 30% higher for the regular and super incontinence pads, respectively. The preliminary outcome of this study again confirmed the clinical acceptability of use for the SDS despite the accuracy data.

### Limitations

However, there were a number of limitations in this study, including the low population number and the brief study duration. Since this was an initial study that examined how well the SDS actually measures urine weight, a large-scale multicenter study is needed for future clinical use. This study may have failed to familiarize health care providers with digital technology, and the period of use of the SDS may have been too short to allow the providers to recognize dermatitis or bedsores in the area of the incontinence pad. In particular, considering the SDS use time per person, it seems important to set the overall study period to at least 1 year in future studies. In addition, since most of this study was conducted with older adults with various diseases, there is a possibility that there may have been differences depending on the disease group. Therefore, future research must expand to include relatively young patients or children.

Our research results show that the reason for the high accuracy ultimately lies in the sensor’s measurement method. The urination state can be detected, but the amount of change in the wetness state is small, and urination cannot be detected if it is outside the set value range, for example, if the reference value (eg, 100) for factors such as impedance, resistance, and capacitance is set in advance, and the value for comparison for currently detected wetness is set to the reference value plus 10 or more (eg, 110). Although urination cannot be detected, our technology detects the wetness state in the diaper in 15-second increments, so precise data can be obtained regardless of whether or not there is a change in wetness (Table 3).

**Table 3:** Comparison of sensing technology among different smart diapers.

	This study	Cho et al [22]	Huion et al [23]
Minimum urine detection amount	1 mL	50 mL	Unknown
Reminder when to change diapers	Diaper change recommendation notification provided when urination volume is predicted to reach 250 mL Provides visualization of monitoring Acoustic alert sound available	None	Unknown
Real-time urination volume prediction	Real-time confirmation with graph scale changes	None	None
Urine detection notification	Alarm from website, app, or both	Alarm from app	Alarm from app
Notification to change diapers	Alarm from website, app, or both	None	None
Urine detection time interval	24-hour detection in real time	Every 10-20 minutes	Detected only when an event occurs
Data transmission interval	1 minute	When an event occurs	When an event occurs
Urine detection rate	100%	32.8%	Unknown
Check for urination pattern	Possible (visualization)	None	None
Role of sensors	Sensors send urination detection data to server	Urine detection event analysis	Urine detection event analysis
System role	Analysis of collected urination detection data Conversion of analyzed urination detection data to visualization content Urine pattern analysis Analysis of use status Automatic urination log recording	None	None

### Conclusions

This study demonstrates the possibility that heath care providers could be alerted by the SDS of the need for pad replacement due to incontinence, thus avoiding the development of dermatological complications. High values for ICC and SDS accuracy were reported in this study, which enabled the mean and total urination volumes of the study participants to be observed. Additional research is merited in order to determine urination habits in older people who require incontinence pads, as well as to evaluate the use of SDSs to identify relevant clinical conditions, such as dehydration, urinary tract infections, or urine retention.

## Appendix

Multimedia Appendix 1 Strengthening the Reporting of Observational Studies in Epidemiology (STROBE) checklist.

## Abbreviations

AWS: Amazon Web Services
FVC: frequency volume chart
ICC: intraclass correlation coefficient
IoT: Internet of Things
LOA: limit of agreement
LUTS: lower urinary tract symptoms
SDS: smart diaper system
SMD: standardized mean difference
STROBE: Strengthening the Reporting of Observational Studies in Epidemiology

## Data Availability

The data sets generated during and/or analyzed during this study are available from the corresponding author on reasonable request.
